# Sterility Remedied

**Published:** 1857-04

**Authors:** 


*Sterility Remedied.*—A correspondent of the New Orleans News and Hospital Gazette, directed a negro woman to apply an infant to her breasts with a view to cure her of sterility. The prescription was followed for only four successive days, during which time she conceived, and at the end of full term was delivered of a fine child. This is another evidence of the remedial use to be made of the close sympathy existing between the mammæ and the uterus and ovaries. Wishing to avail ourselves of this means for the relief of amenorrhœa, lately, we used the gum elastic cup as very convenient of application, and with good effect. This was done after sinapisms had failed.—*Memphis Med. Recorder.*

